# Validity and reliability of smartphone applications for measurement of hip rotation, compared with three‐dimensional motion analysis

**DOI:** 10.1186/s12891-021-03995-2

**Published:** 2021-02-11

**Authors:** Phob Ganokroj, Nuchanun Sompornpanich, Pichitpol Kerdsomnuek, Bavornrat Vanadurongwan, Pisit Lertwanich

**Affiliations:** Department of Orthopaedic Surgery, Faculty of Medicine Siriraj Hospital, Mahidol University, 2 Prannok Road, Bangkok Noi, 10700 Bangkok, Thailand

**Keywords:** Smartphone application, Hip rotation, Three-dimensional motion analysis, Measurement, Validity, Reliability

## Abstract

**Background:**

Measurement of hip rotation is a crucial clinical parameter for the identification of hip problems and the monitoring of symptoms. The objective of this study was to determine whether the use of two smartphone applications is valid and reliable for the measurement of hip rotation.

**Methods:**

An experimental, cross-sectional study was undertaken to assess passive hip internal and external rotation in three positions by two examiners. The hip rotational angles were measured by a smartphone clinometer application in the sitting and prone positions, and by a smartphone compass application in the supine position; their results were compared with those of the standard, three-dimensional, motion analysis system. The validities and inter-rater and intra-rater reliabilities of the smartphone applications were evaluated.

**Results:**

The study involved 24 participants. The validities were good to excellent for the internal rotation angles in all positions (ICC 0.81–0.94), good for the external rotation angles in the prone position (ICC 0.79), and fair for the sitting and supine positions (ICC 0.70–0.73). The measurement of the hip internal rotation in the supine position had the highest ICC value of 0.94 (0.91, 0.96). The two smartphone applications showed good-to-excellent intra-rater reliability, but good-to-excellent inter-rater reliability for only three of the six positions (two other positions had fair reliability, while one position demonstrated poor reliability).

**Conclusions:**

The two smartphone applications have good-to-excellent validity and intra-rater reliability, but only fair-to-good inter-rater reliability for the measurement of the hip rotational angle. The most valid hip rotational position in this study was the supine IR angle measurement, while the lowest validity was the ER angle measurement in the sitting position. The smartphone application is one of the practical measurements in hip rotational angles.

**Trial registration:**

Number 20181022003 at the Thai Clinical Trials Registry (http://www.clinicaltrials.in.th) which was retrospectively registered at 2018-10-18 15:30:29.

## Background

The hip joint is a ball and socket joint formed by an articulation between the femoral head and the acetabulum, which has a wide range of motion. Measurement of hip movement is a crucial clinical parameter for the identification of hip problems and the monitoring of clinical progression after treatment. Six common hip movements in three planes are usually evaluated during physical examination: flexion, extension, abduction, adduction, internal rotation (IR), and external rotation (ER). Of these, loss of IR is a common presentation of patients who have hip problems such as an osteoarthritic hip or femoroacetabular impingement (FAI) [1–3]. Varying degrees of IR deficit in the hip were found in asymptomatic adolescent athletes; two-thirds of the study cohort had radiographs suggestive of FAI [4].

Hip examinations can be performed in various positions, such as the supine, prone, or sitting positions. There is no gold standard for the best position for the measurement of each hip motion. In previous studies, the hip range of motion was measured using a range of methods, such as visual estimation, a manual goniometer [4], photographic measurement [5], a digital inclinometer [6], and three-dimensional motion analysis (3DMA) [7]. However, there are differences in the measurements provided by the various traditional methods, especially when estimating IR and ER [6]. In addition, smartphone applications are now available to measure hip motion [8]. The objective of this study was to ascertain whether the use of two particular smartphone applications is valid and reliable for the measurement of hip rotation, compared with a three-dimensional motion analysis. The hypothesis was that the two smartphone applications are alternative instruments that provide acceptable validity and reliability for hip rotational angle measurements.

## Methods

This cross-sectional study was approved by the Institutional Review Board. Prior to its commencement, all participants read an information sheet and gave written, informed consent. The research was registered with the Thai Clinical Trials Registry (http://www.clinicaltrials.in.th; registration number TCTR20181022003). The study was performed at a motion analysis laboratory and sports clinic at our hospital. The inclusion criteria were participants aged between 20 and 50 years who had a body mass index (BMI) of less than 30 kg/m^2^. The exclusion criteria were: (1) a prior history of hip or knee pain; (2) previous hip or knee surgery; and (3) a previous hip or other lower-extremity fracture.

### Intervention

#### Smartphone applications

Two smartphone applications were utilized for this study: (1) “Bubble Level and Clinometer” by Peter Breitling, for vertical-plane measurements (App Store: https://appsto.re/th/cdqis.i; Google Play: https://play.google.com/store/apps/details?id=com.plaincode.clinometer); and (2) “Accurate Compass” by Ngo Na, for horizontal-plane measurements (App Store: https://appsto.re/th/LQmW_.i; Google Play: https://play.google.com/store/apps/details?id=com.dungelin.compass). The smartphone used in this study was an iPhone 6 with an iOS 9 operating system. The measurements were conducted on the right hip of the participants. The smartphone was securely attached with a smartphone armband to the right leg at the level of the tibial tubercle and in the axis of the anterior tibia crest. However, in the event that a participant’s leg proved too large to allow us to secure the phone with the armband, rigid tapes were used to firmly attach the device to the leg.

#### Three‐dimensional motion analysis

Eight optoelectronic cameras (Motion Analysis Corp., Santa Rosa, CA, USA) were used to record three-dimensional kinematic data at a sampling rate of 200 Hz. Thirteen reflective markers were attached at the following anatomical landmarks: both anterior superior iliac spines (ASIS); the distal lateral femoral condyle; the midpoint between the imaginary line from the ASIS to the lateral distal femoral condyle; the distal medial femoral condyle; the midpoint between the imaginary line from the ASIS to the distal medial femoral condyle; the medial and lateral malleoli; and the sacrum. Modeling of the anatomical segments, especially the thigh and leg compartments, was created on a computer program. The angles of the lower leg at the starting point and end point of motion were calculated as the IR and ER angles, respectively. For the purposes of this study, 3DMA was considered as the gold standard for hip rotation angle measurement.

#### Measurement techniques

To assess the validities and reliabilities of the two smartphone applications, the same protocol was followed for the measurements of the right hip rotations of the participants. The rotations were assessed by two examiners: a sports medicine orthopedic surgeon, and an orthopedic resident who had previously trained for the hip rotation examination before conducting this study. The participants’ hips were passively rotated internally and externally by the examiners until the end points were reached; each procedure, and the related measurement that was taken, was repeated three times. The end point of each measurement was defined as when the examiners either detected a firm- or stiff-end feeling, or observed a compensatory movement at the pelvis and/or trunk. The hip rotations were measured in the following three positions:

1. A sitting position. The participants sat on the edge of a table with their right hip and right knee flexed to 90 degrees. The participants’ right knee was fixed with one examiner’s hand while their right ankle was passively moved laterally and medially by the other examiner’s hand (Fig. 1a and b). The Bubble Level and Clinometer mobile application was employed to measure the rotation angles. The angle at the starting point—the neutral position—was recorded. Then, the angle was noted again once the end points of the passive internal and external hip rotations were reached. The difference between the starting and end points gave the rotational angle of the right hip in each direction.

**Fig. 1:**
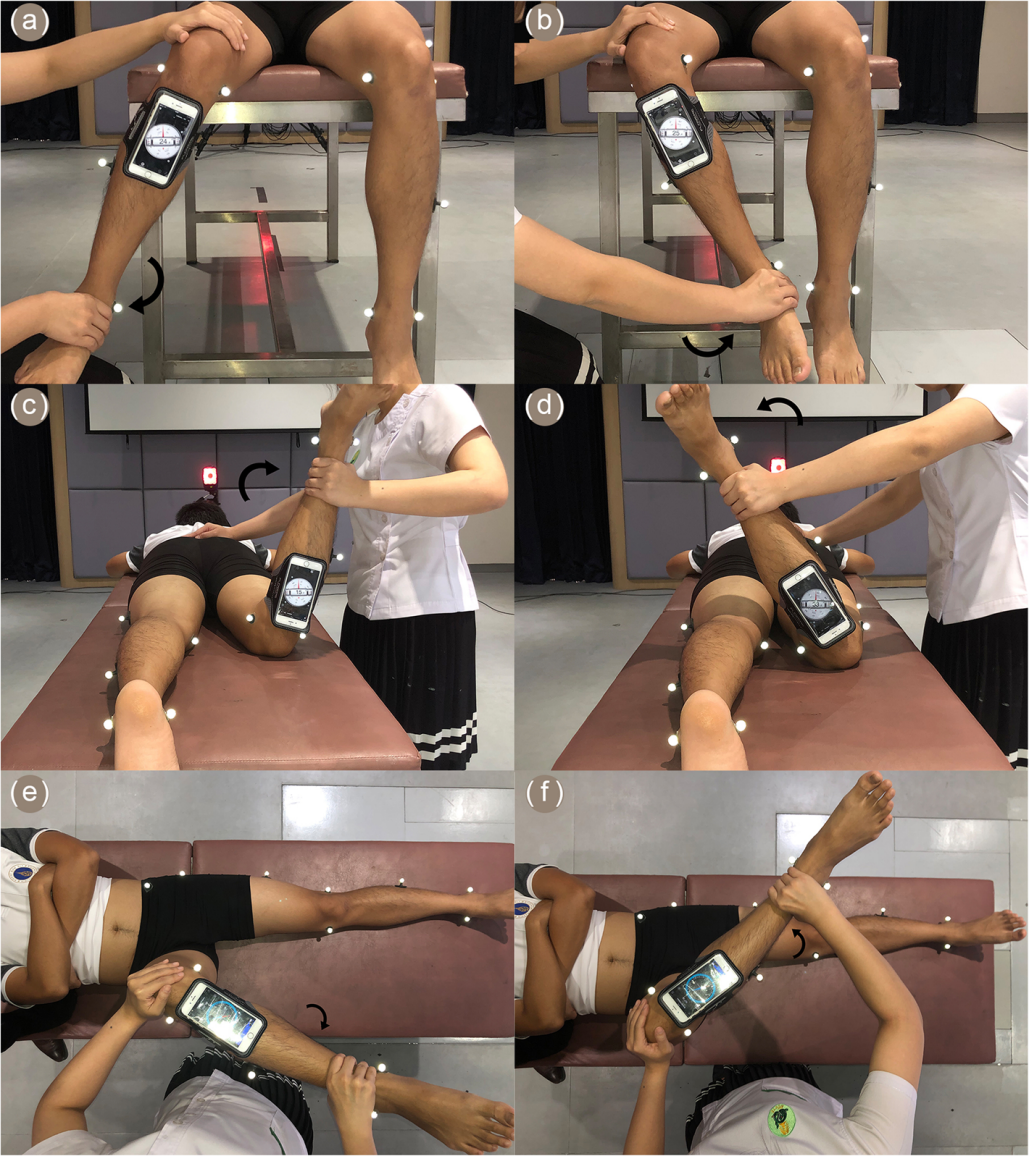
Measurement technique of smartphone applications to evaluate passive hip rotational angles in three positions: **a** sitting hip internal rotation; **b** sitting hip external rotation; **c** prone hip internal rotation; **d** prone hip external rotation; **e** supine hip internal rotation; and **f** supine hip external rotation
2. A prone position. The participants lay face down with their right knee flexed to 90 degrees while their left leg was placed flat on the table. The examiners held the right ankle with one hand while a second hand fixed the participants’ right knee on the table. Afterwards, the participants’ right ankle was move laterally and medially to represent the internal and external rotation angles of the hip, respectively (Fig. 1c and d). Again, the Bubble Level and Clinometer mobile application was utilized to measure the rotation angles in these positions. The difference between the starting and end positions represented the rotation angle of the right hip in the prone position.
3. A supine position. The participants lay on the examination table with their right hip and knee passively flexed to 90 degrees. The examiners held the participants’ right ankle with one hand and the participants’ right knee with another hand. With the right knee fixed, the participants’ right ankle was then moved laterally and medially from the neutral position until it reached the end point. The lateral and medial angles represented the IR and ER angles of the right hip, respectively (Fig. 1e and f). The mobile application Accurate Compass was selected to determine the rotation angle in the horizontal plane. The rotation angle calculation was conducted in the same manner as for the sitting and prone positions

On the first day of the measurements, the 3DMA assessments of the hip rotations as well as the smartphone-based calculations were performed by both examiners. One-week later, the measurements were repeated by the 2 examiners using the same protocol, but only the smartphone applications were used (to assess the inter- and intra-rater reliabilities), not the 3DMA.

### Statistical analysis

The estimated sample size was calculated based on the primary outcome of the reliability of measurement, the intraclass correlation coefficient (ICC); the estimate of the ICC was set at 0.8 ± 0.1 [8]. Given that estimate, having 24 patients would allow the achievement of 95 % of CI [9].

Descriptive statistics were used to summarize the demographic data. The validities and inter-rater and intra-rater reliabilities of the smartphone applications were assessed via ICC, standard error of measurement (SEM), and limit of agreement (LOA). Based on the 95 % CI of the ICC, the reliability values were classified as excellent (ICC ≥ 0.9); good (ICC 0.75–0.89); fair (ICC 0.5–0.75); and poor (ICC < 0.5) [10]. Using Bland–Altman plots and scatter plot graphs, SEM and LOA were presented as indicators of absolute reliability. Statistical analyses were performed using SPSS Statistics for Windows, version 18.0 (SPSS Inc., Chicago, IL, USA). The level of significant difference was set at 0.05.

## Results

The study included 24 participants. They had a mean age of 26.1 ± 4.8 years and a mean BMI of 22.6 ± 3.3 kg/m^2^, and 10 were male. The mean ranges of the hip rotation motions for each position are listed in Table 1. For all positions, the values of the hip IR angles were lower than those for the ER angles, and the hip rotation values determined by the smartphone applications were greater than those measured by the 3DMA (*p*-value < 0.01). Table 2 details the validities of the two smartphone applications against the 3DMA in terms of ICC (95 % CI), SEM, % SEM, and LOA values. The ICC values of the smartphone applications provided good-to-excellent results for the IR angles in all positions (ICC 0.81–0.94). There were also good results (ICC 0.79) for the ER angles in the prone position, but only fair results (ICC 0.70–0.73) for the sitting and supine positions. The ICC values of the IR angles were generally higher than those for the ER angles in the same position. The measurement of the hip IR in the supine position showed the highest ICC value (0.94), whereas the hip ER in the sitting position had the lowest ICC value (0.70).

**Table 1:** Mean hip rotation angles for each position measured by the smartphone applications and the three-dimensional motion analysis

Positions	Smartphone Mean ± SD (degrees)	3DMA Mean ± SD (degrees)
Sitting IR^a^	29.4 ± 7.5	19.7 ± 6.9
Sitting ER^a^	36.4 ± 5.3	29.4 ± 4.7
Prone IR^a^	35.3 ± 10.2	29.9 ± 8.2
Prone ER^a^	43.4 ± 8.9	35.0 ± 7.2
Supine IR^b^	31.4 ± 9.0	26.5 ± 7.9
Supine ER^b^	52.3 ± 7.7	40.6 ± 7.4

*3DMA* three-dimension motion analysis; *IR* internal rotation; *ER* external rotation

^a^ Measured by The Bubble Level and Clinometer

^b^ Measured by The Accurate Compass

**Table 2:** Validity of the smartphone applications versus the 3DMA for each position

Position	ICC (lower, upper)	SEM (°)	%SEM	LOA
Sitting IR^a^	0.89 (0.83, 0.93)	2.9	11.7	(16.5, 3.1)
Sitting ER^a^	0.70 (0.56, 0.80)	3.5	10.7	(15.2, -1.0)
Prone IR^a^	0.81 (0.71, 0.86)	4.3	13.1	(16.9, -6.1)
Prone ER^a^	0.79 (0.68, 0.86)	4.2	10.7	(18.7, -2.2)
Supine IR^b^	0.94 (0.91, 0.96)	2.2	7.5	(10.7, -0.9)
Supine ER^b^	0.73 (0.60, 0.82)	5.0	10.8	(22.8, 0.5)

*IR* internal rotation; *ER* external rotation; *ICC* intraclass correlation coefficient; *SEM* standard error of measurement; *LOA* limit of agreement

^a^ Measured by The Bubble Level and Clinometer

^b^ Measured by The Accurate Compass

The intra-rater reliabilities of the smartphone applications for Rater A and Rater B were presented in Table 3. Both Rater A and B showed good-to-excellent intra-rater reliabilities for all positions. As to the IR angle measurements in the supine position, both raters had an excellent intra-rater reliability (ICC 0.93). With regard to the inter-rater reliabilities of the smartphone applications listed in Table 4, the ICC value demonstrated excellent results for the IR angle measurements in the supine position (ICC 0.90), good results in the prone position (ICC 0.81), but fair results in the sitting position (ICC 0.67). By comparison, there was good inter-rater reliability for the ER angle measurements in the prone position (ICC 0.76), a fair result for the sitting position (ICC 0.56), and a poor result for the supine position (ICC 0.43). There were more inter-rater reliabilities for the IR-angle measurements than the ER-angle measurements in all positions.

**Table 3:** Intra-rater reliability measured by the smartphone applications and the 3DMA for each position

Positions	Rater A				Rater B			
	***ICC (lower, upper)***	***SEM (°)***	***%SEM***	***LOA***	***ICC (lower, upper)***	***SEM (°)***	***%SEM***	***LOA***
Sitting IR^a^	0.97 (0.94, 0.99)	1.33	4.5	(-2.4, 4.6)	0.87 (0.71, 0.94)	0.78	3.2	(-5.6, 3.6)
Sitting ER^a^	0.89 (0.77, 0.95)	1.85	5.1	(-4.9, 5.2)	0.93 (0.84, 0.97)	1.19	3.9	(-4.1, 2.6)
Prone IR^a^	0.91 (0.81, 0.96)	3.22	9.3	(-11.2, 6.2)	0.87 (0.72, 0.94)	3.04	7.6	(-11.0, 5.8)
Prone ER^a^	0.90 (0.78, 0.95)	2.8	6.4	(-8.1, 7.7)	0.88 (0.74, 0.95)	2.59	5.6	(-7.8, 6.7)
Supine IR^b^	0.93 (0.84, 0.97)	2.48	7.8	(-7.4, 6.8)	0.93 (0.84, 0.97)	2.18	6.4	(-8.8, 3.4)
Supine ER^b^	0.88 (0.74, 0.95)	2.77	5.3	(-6.5, 9.1)	0.85 (0.69, 0.93)	3.68	7.0	(-11.9, 8.4)

*IR* internal rotation; *ER* external rotation; *ICC* intraclass correlation coefficient; *SEM* standard error of measurement; *LOA* limit of agreement

^a^ Measured by The Bubble Level and Clinometer

^b^ Measured by The Accurate Compass

**Table 4:** Inter-rater reliability measured by the smartphone applications for each position

Positions	ICC (lower, upper)	SEM (°)	%SEM	LOA
Sitting IR^a^	0.67 (0.37, 0.84)	3.4	12.7	(-4.8, 14.7)
Sitting ER^a^	0.56 (0.20, 0.78)	3.3	9.9	(-3.8, 14.7)
Prone IR^a^	0.81 (0.71, 0.94)	4.1	10.7	(-14.6, 4.7)
Prone ER^a^	0.76 (0.52, 0.89)	3.9	8.8	(-14.4, 7.7)
Supine IR^b^	0.90 (0.79, 0.96)	2.7	4.8	(-9.9, 4.6)
Supine ER^b^	0.43 (0.04 0.71)	2.6	8.1	(-23.2, 12.1)

*IR* internal rotation; *ER* external rotation; *ICC* intraclass correlation coefficient; *SEM* standard error of measurement; *LOA* limit of agreement

^a^ Measured by The Bubble Level and Clinometer

^b^ Measured by The Accurate Compass

**Fig. 2:**
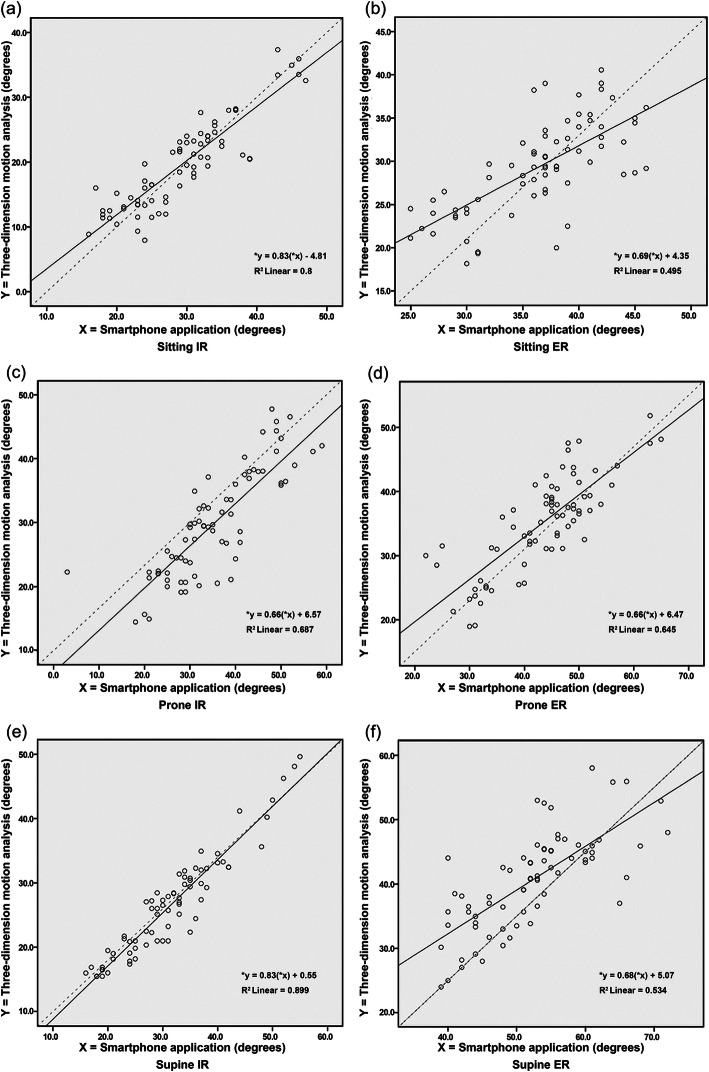
Scatter plot of passive hip rotation angles measured by three-dimensional motion analysis and the smartphone applications. Each data point is the average of the rotation angle of the two measurements in different positions: **a** sitting hip internal rotation; **b** sitting hip external rotation; **c** prone hip internal rotation; **d** prone hip external rotation; **e** supine hip internal rotation; and **f** supine hip external rotation

**Fig. 3:**
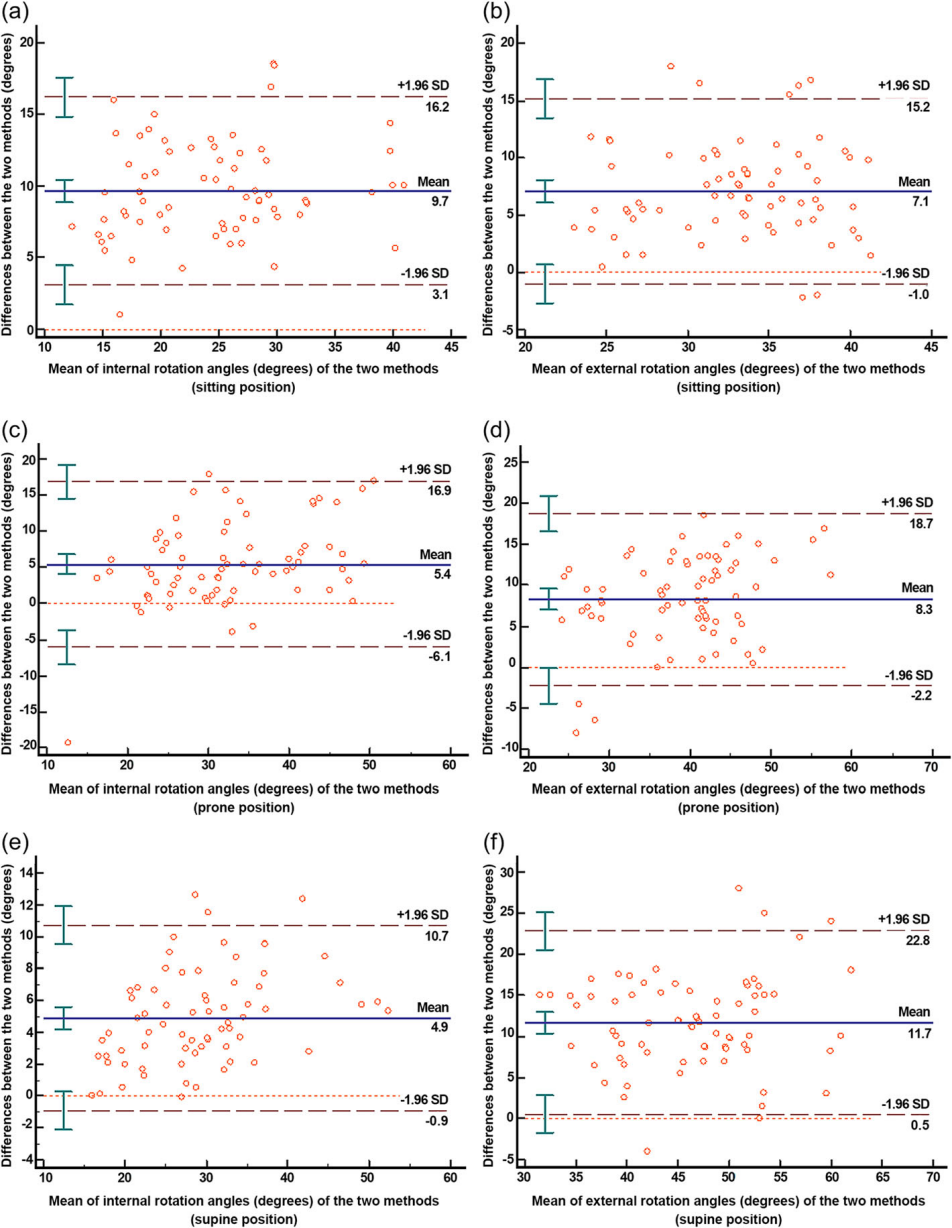
Bland–Altman plots. Comparison of the difference between the two methods of measurement (smartphone applications and three-dimensional motion analysis system) versus the mean of the two methods. The mean difference is given by the solid line. The limits of agreement are given by the ± 2SD limits: **a** sitting hip internal rotation; **b** sitting hip external rotation; **c** prone hip internal rotation; **d** prone hip external rotation; **e** supine hip internal rotation; and **f** supine hip external rotation

The mean rotation angles from the 3DMA and the smartphone applications were plotted on the scatter plot graphs for each position (Fig. 2). The residual plot and the R^2^ value for the regression model showed the smallest differences in the rotation angles for the IR angle measurements in the supine position (R^2^ = 0.89). The differences between the rotation angle obtained with the two measurements (3DMA and smartphone application) versus the means of those measurements are illustrated by the Bland–Altman plot graphs for all positions in Fig. 3. The overall LOA of the IR angle measurements were smaller than the ER angle measurements for all positions (Fig. 3).

## Discussion

This study focused on whether the smartphone applications can demonstrate the true hip rotational angle (validity) and produce constant results (reliability). The major findings in the study are that the smartphone applications mostly showed good-to-excellent validity compared with the 3DMA measurements. The most valid hip rotational position in this study was the supine IR angle measurement, while the lowest validity was the ER angle measurement in the sitting position. In addition, the study found good-to-excellent inter- and intra-rater reliabilities for the smartphone application measurements. Our findings are similar to those of a study by Charlton et al. [8]; they investigated a smartphone application called “Hip ROM Tester” (https://play.google.com/store/apps), which was mainly used for calculations in its clinometer-like mode. Those researchers found that, compared with 3DMA, the Hip ROM Tester application demonstrated excellent validity for all hip motions except the supine ER position, which only had moderate-to-good validity. The research team measured the rotational angles in two positions (sitting and supine); however, the knee was placed off the examination table and flexed to 90 degrees, which is not a common posture in routine clinical practice [8]. In the current study, however, the authors set up the supine position so that the participants lay on the table, with their hip and knee being passively flexed to 90 degrees; this is a more typical position for the assessment of hip rotations [3].

Moreover, as done in earlier research by Tousignant-Laflamme et al., the rotational angles in the present study were assessed by two mobile applications, the choice of which varied with the specific plane of measurement [11]. The authors believe that using both an inclinometer and a gyroscope-like mobile application may be more appropriate than relying solely on one application. The recent studies support mobile applications in determining hip rotational angles. The smartphone compass application could quantify the hip IR angles in a supine position with good intra-rater and moderate inter-rater reliabilities [12]. Active hip IR and ER could be measured with the clinometer smartphone applications [13]. The smartphone accelerometer-based goniometers had good validity compared with computer assist navigation systems with less than 3-degree acceptable clinical error [14].

All values from the application measurements were higher than those from the 3DMA, with a p-value of less than 0.01. The same results had been found by a previous study that compared the validity of a manual goniometer with an electromagnetic tracking system [15]. In that research, it was found that the rotational angles obtained from the manual goniometer did not indicate the true hip rotation movement. Instead, the values from the manual goniometer represented the intersegment of the thigh and/or leg to the trunk and/or the table angle [15]. Such measurements may include pelvic tilt and/or lumbar movement. In the case of the 3DMA, however, the angles were calculated by a computer based on anatomical landmarks attached to the skin. For that reason, the results from the smartphone application were higher than those from the standard 3DMA measurement in the current study.

The authors found good-to-excellent intra-rater reliabilities for both examiners in this study. As to the inter-rater reliabilities, there were good-to-excellent reliabilities for three of the six positions; another two had a fair reliability, while the last one (the supine ER position) had a poor reliability. The best intra-and inter-rater reliabilities were found with supine IR. The authors believe the cause of the poor-to-fair inter-rater reliability was related to technical errors in the measurements rather than any inaccuracy of the mobile application. There were inconsistent end-point measurements despite the study clearly defining the end point. The precision of the measurements might have been affected by the variety of body shapes of the patients as well as by the diversity of body shapes, sex, and ages of the examiners. The authors suggest that smartphone applications should be used with caution. Examiners should be well trained before applying a mobile application in clinical practice.

There are many factors associated with the validity of the measurements. In this study, the validity of the smartphone applications depended on the measurement position, with the supine position having greater validity (the highest ICC 0.94; 7.5 % SEM) than those of both the sitting position (ICC of 0.89; 11.7 % SEM) and the prone position (ICC of 0.81, 13.1 % SEM). Furthermore, the study by Simonneau et al. [16] found that the hip position influenced the measurements of the rotation angle made with a manual goniometer in healthy subjects. The researchers stated that there were more values for ER hip angles measured in the prone position than in the sitting position, and the difference was statistically significant. On the other hand, while the values of the IR hip angles were also higher in the prone position, the difference was not significant [16]. In contrast, work by Kouyoumdjian et al. [5] found that the measurements of the hip rotation angles did not differ significantly for all three positions (supine, prone, and sitting).

To date, there is no consensus regarding which is the best position to measure hip rotation. The authors noted that it was hard to limit pelvic movements and lift the hip while performing the hip rotation measurement in the supine and prone positions. For the supine position in this study’s protocol, the right hip and pelvis were lain and fixed on the examination table. The right hip and knee were flexed to 90 degrees, and the right knee was stabilized through rotational movement. This protocol might minimize pelvic movements and thus reduce errors of measurement. The authors suggest that there are differences in the measurement of the hip rotation in different positions. The supine position may be the optimal position to measure the hip rotational angle.

The present study found that the IR hip measurements had greater validity than the ER hip measurements. This was similar to the findings of the work by Nussbaumer et al. [15], who investigated the validity of manual goniometers for the measurement of passive hip rotations in FAI patients. As with our study, those researchers identified that there was a higher validity for the measurement of passive IR angles than of ER angles [15]. Another study has also found that the bubble inclinometer has greater validities and intra-rater reliabilities for the measurements of IR angles than ER angles [8]. In addition, smartphone applications have been used in a number of studies to measure joint angle motions [17–20]. Compared with the universal goniometer, the applications showed good-to-excellent agreement and good reliability for the measurement of many joint angle motions of the knee [17], ankle [18], and wrist joints [19]. For that reason, the smartphone applications in the current study might be used to assess the hip rotational angle.

The smartphone applications, Bubble Level and Clinometer and Accurate Compass, offer many advantages: (1) a low cost, (2) convenience, (3) quick assessments, (4) a user-friendly operation, and (5) acceptable validity and reliability. Moreover, an examiner could use the applications without the aid of an assistant. To do this, the examiner would use one hand to rotate the hip and the other hand to stabilize the pelvis. The rotation angles would be estimated by the examiner’s eyes or measured by an assistant using a manual goniometer.

This study had some limitations. Firstly, the end points of each measurement were subjective. The authors clearly stated the end point as being either the appearance of a firm-end feeling or the observation of pelvic and/or trunk movements. Nevertheless, this particular protocol might create inconsistency in determining the end points by individual examiners. It would therefore be better if either the pelvic tilt or rotation was utilized as the objective measurement to reduce errors. Secondly, the 3DMA angle measurements depended on the reflexive anatomical landmarks applied on the skin. It would be better if we applied the marker on the bone in order to obtain a more precise angle measurement. Unfortunately, that could not be done in routine clinical practice. Finally, the smartphone was attached to the participant by an armband. This may create some soft tissue artifact due to micro-motion, especially in the case of large leg sizes. The authors attempted to solve this problem by using rigid tape. Moreover, the measurements were made three times each to assess the intra-rater reliability; however, measurement errors might still occur. The likelihood of their occurring would be reduced in a future study if a better way of securing the mobile phone to the participants’ skin is devised or employed. At this time, development in machine learning technology have gained popularly especially for the musculoskeletal system to solve the clinical problem [21]. A better mobile application system together with the machine learning technology may have benefit in clinical practice in the future.

## Conclusions

The smartphone applications have good-to-excellent validity and intra-rater reliability, but fair-to-good inter-rater reliability for the measurement of hip rotational angles. The most valid hip rotational position in this study was the supine IR angle measurement, while the lowest validity was the ER angle measurement in the sitting position. The smartphone application is one of the practical measurements in hip rotational angles.

## Data Availability

The datasets used and/or analyzed during the current study are available from the corresponding author on reasonable request.

## Abbreviations

ICC: The intra-class correlation coefficient
IR: Internal rotation
ER: External rotation
FAI: Femoroacetabular impingement
3DMA: Three-dimension motion analysis
ASIS: Anterior superior iliac spine
SEM: Standard error of measurement
LOA: Limit of agreement
BMI: Body mass index
